# Dynamic receptive field and feature enhanced network for bridge defect detection

**DOI:** 10.3389/frai.2026.1952072

**Published:** 2026-10-28

**Authors:** Ziqi Tang, Yuang Ji, Bin Chen, Shan Huang, Yan Jin, Jichao Liu, Hui Li

**Affiliations:** 1 China Harbour Engineering Company Limited, Beijing, China; 2 Qingdao University of Science and Technology, Qingdao, Shandong, China; 3 Hainayun IoT Technology Ltd., Qingdao, Shandong, China

**Keywords:** bridge defect detection, dynamic detection head, linear attention, multi-scale object detection, spatial channel downsampling, switchable dilated convolutions

## Abstract

**Introduction:**

Accurate defect detection in underwater and cross-domain marine infrastructure, such as bridge piers, submarine pipelines, and subsea equipment, is essential for structural safety assessment and intelligent ocean engineering. Existing methods, however, suffer from large scale variations, dense small targets, severe class imbalance, and texture loss in complex underwater environments.

**Methods:**

We propose a multi–dimensional collaborative detection network based on YOLOv10n. In the backbone, switchable dilated convolutions (SAConv) adaptively adjust receptive fields, and a spatial-channel downsampling (SCDown) module preserves fine-grained details. At the backbone output, a Partial Spatial Attention and Multi-layer Latent Linear Attention (PSAMLLA) module captures global context with linear complexity via rotary and local position encoding. In the feature pyramid, a Conditional Identity Block Feature Fusion (C2fCIB) module enhances multi-scale fusion using deep large-kernel convolutions with structural re-parameterization. Finally, a Dynamic Detection Head (DyHead) integrates scale-aware, spatial-aware, and task-aware attention for adaptive inference.

**Results:**

Extensive experiments on the public bridge-bkqxz/2 dataset demonstrate that our method achieves comparable or better results than several state-of-the-art detectors while maintaining low computational cost.

**Discussion:**

Although the current experiments are limited to bridge surface defect detection, the proposed techniques address challenges such as texture degradation and multi-scale object variability and may provide a useful foundation for future extension to underwater vision systems, which remain an important direction for subsequent investigation.

## 1 Introduction

Bridges—as well as marine infrastructures such as offshore platforms, submarine pipelines, and underwater tunnel components—are critical to global transportation and energy networks (ZHANG et al., 2022; Ye et al., 2020; Medvediev et al., 2024; Nemine and Orie, 2025; Amirkhani et al., 2024). Bridges structural safety is directly linked to public safety and the smooth operation of regional economies. With increasing service years, bridge structures inevitably develop various surface defects—including cracks, spalling, water infiltration, corrosion, and exposed rebar—under the combined effects of loading, environmental erosion, and material aging. If not detected and addressed promptly, these defects may progressively evolve into severe structural damage and even lead to catastrophic accidents. Therefore, efficient and accurate bridge defect detection constitutes an essential technical means of ensuring structural safety and enabling scientific operation and maintenance management. Traditional manual inspection of these structures suffers from subjectivity, low efficiency, and inaccessibility, particularly in hazardous or underwater environments. While the present study focuses on bridge surface defect detection, the technical challenges addressed such as texture degradation and multi-scale object variability—share conceptual similarities with underwater inspection scenarios. With advances in deep learning, computer vision has emerged as a promising alternative for automated defect detection. Recent efforts have explored attention mechanisms (Ruggieri et al., 2025), wavelet transform with CNNs (Yu et al., 2025), graph neural networks (He et al., 2024), multi-scale dynamic heads (Luo et al., 2025), and Transformer-based segmentation (Flotzinger et al., 2024) to improve accuracy.

However, existing deep learning-based bridge defect detection methods still face several critical challenges in practical applications, which severely constrain their detection performance and engineering deployment. First, bridge defects exhibit extreme scale variations—fine cracks may be only millimeters in width, while large-area spalling and water infiltration zones can extend to several meters. Standard convolutions in conventional detection networks have fixed receptive fields, making it difficult to simultaneously capture global semantic information for large-scale defects and local texture details for small-scale defects. Second, many bridge defect targets, such as cracks and early-stage rust spots, are typical small objects whose texture and edge information are easily overwhelmed by background noise after multiple down-sampling operations, resulting in significantly low recall rates for small-target detection. Third, the sample sizes of different defect categories in bridge defect datasets are severely imbalanced—common defects such as cracks and efflorescence possess tens of thousands of samples, while rare defects such as corrosion, deep cracks, and formwork niches have only a few hundred. This long-tail distribution causes model training to be heavily biased toward high-frequency categories, leading to substantially degraded detection performance for low-frequency classes. Fourth, bridge structures often present complex background textures—including formwork seams, water stains, shadows, and graffiti—which are visually similar to real defects, making it difficult for models to distinguish defects from background interference and resulting in high rates of false positives and missed detections. Wu and Kundu (2026) review recent advances in physics-guided, physics-informed and physics-encoded machine learning for condition monitoring. Although its focus is not limited to corrosion images, it provides a methodological framework for incorporating physical a priori knowledge into data-driven defect detection, alleviating data scarcity and enhancing interpretability. Yu et al. (2026) conduct a benchmark study on deep learning for binary classification in the context of marine corrosion, construct a comprehensive marine corrosion dataset, and validate the feasibility of deep learning for automated corrosion detection. Chliveros et al. (2024) propose a deep learning-based image corrosion classification method utilizing the Eigen Tree Hierarchy module, designed for the identification of corroded areas on the anti-corrosion coatings and surfaces of marine vessels. Alharbi et al. (2026) place particular focus on detecting hidden corrosion in galvanized steel, which requires specialized sensors and AI models that can pick up subtle changes in the material. Chliveros et al. (2023) propose an unsupervised method for eigen tree hierarchies and quantisation group association for segmentation of corrosion in marine vessel hull inspection via camera images. These four challenges are mutually coupled, severely limiting the performance of generic object detectors when directly applied to bridge defect detection tasks. Beyond the specific technical challenges in bridge defect detection, these difficulties echo broader issues identified in the AI for Science literature, including the scarcity of standardized high-quality data and the persistent interpretability gap between model predictions and physically meaningful explanations (Xu et al., 2026). Addressing these fundamental challenges is essential for deploying AI-based defect detection systems in real-world engineering practice.

To address the above issues, we propose a multi-dimensional collaborative detection network based on the YOLOv10n architecture. Specifically:

1. In the backbone, we replace standard convolutions with switchable dilated convolutions (SAConv) to adaptively adjust receptive fields, and introduce a spatial-channel downsampling (SCDown) module to decouple channel transformation from spatial reduction, thereby preserving small-defect textures.
2. At the backbone end, we design a Partial Spatial Attention and Multi-layer Latent Linear Attention (PSAMLLA) module, which models global context with linear complexity using a dual-branch partial attention design, enhanced by rotary and local position encodings for robust spatial awareness.
3. In the deepest feature pyramid fusion path, we incorporate a Conditional Identity Block Feature Fusion (C2fCIB) module, built upon full-depth convolutions and RepVGG-style large kernels via structural re-parameterization, to improve multi-scale fusion without extra inference cost.
4. We adopt a Dynamic Detection Head (DyHead) that collaboratively applies scale-aware, spatial-aware, and task-aware attention mechanisms for adaptive inference.

Starting from the core information bottlenecks of bridge defect detection, we deploy targeted solutions along the information processing pipeline in a cascaded manner. Specifically, SAConv adaptively adjusts the receptive field at the front-end of the backbone to handle large-scale variations; SCDown decouples spatial downsampling from channel transformation at the mid-end to preserve fine-grained textures of small targets; PSAMLLA models global context with linear complexity at the backbone output to compensate for the lack of spatial-semantic associations in deep features; C2fCIB enhances cross-scale feature fusion in the neck path to improve the interaction quality between shallow and deep features; and DyHead distinguishes classification and regression task requirements at the detection head to resolve task-conflicting feature demands.

## 2 Related work

### 2.1 Methods for detecting bridge defects

Traditional bridge defect detection relies primarily on manual inspections, which are inefficient, highly subjective, and unable to meet the daily maintenance and operational requirements of large-scale bridge networks. In recent years, significant progress has been made in automated defect detection methods based on deep learning. Cha et al. (2017) were the first to apply convolutional neural networks (CNNs) to the detection of surface cracks in concrete, achieving high detection accuracy under limited sample conditions through transfer learning strategies. Xu and Bao (2019) proposed a bridge defect detection framework based on UAV aerial imagery and Faster R-CNN, demonstrating the feasibility of deep learning in bridge inspection scenarios. Since then, Zhang et al. (2021) proposed a detection method based on multi-scale feature fusion to address the issue of significant variations in defect scale. Dung et al. (2019) provided a systematic review of computer vision-based infrastructure inspection methods, identifying small-object detection and class imbalance as the two core challenges facing bridge defect detection. The YOLO series have garnered widespread attention due to their excellent balance between speed and accuracy. Tan and Yang (2024) applied YOLOv5 to bridge crack detection, enhancing the detection accuracy of small cracks by improving the feature pyramid structure. Li et al. (2025) build a new backbone network, DSIBCSPDarknet, which strengthens feature extraction capabilities. The SPPFCSPC structure is introduced to replace the spatial pyramid pooling fast module, enabling more effective multiscale feature fusion. Sun et al. (2025) propose a visual-semantic alignment method for automatic defect detection and diagnosis of prestressed concrete bridges. Xu et al. (2024) augment the network's adaptability to a broad spectrum of bridge defect images through the integration of ODConv into the second convolutional layer, processing information within a four-dimensional kernel space. Luo et al. (2025)improve small-size defects recognition by integrating the high-resolution P2 layer from the backbone into the neck, reducing false detection rates. They also optimize processing, improving defect detection accuracy in multi-scale defects and complex backgrounds. Although the aforementioned work has achieved some success, there remains significant room for improvement in the detection accuracy and generalization capabilities of existing methods when faced with real-world scenarios characterized by a large number of defect categories (over 20), dense distributions of small objects, and complex background interference.

### 2.2 Attention mechanisms and dynamic detection heads

Attention mechanisms effectively enhance a model's ability to focus on key information by adaptively adjusting the weight distribution of feature responses. In terms of feature enhancement, the Feature Pyramid Network (FPN) proposed by Lin et al. (2017) constructs a multi-scale feature pyramid through top-down paths and lateral connections, and has become a standard component of object detection networks. The Squeeze-and-Excitation (SE) network proposed by Hu et al. (2018) achieves adaptive recalibration of feature channels through a channel attention mechanism. Woo et al. (2018) fused channel attention with spatial attention. At the detection head level, Dai et al. (2021) unified the design of different object detection heads by collaboratively introducing three mechanisms—scale-aware attention. The core computational unit of DyHead employs Modulated Deformable Convolution (Zhu et al., 2019), a module that introduces a modulation scalar mechanism based on the previous generation of deformable convolutions (Dai et al., 2017), enabling the network to adaptively adjust the intensity of feature responses at different sampling positions. In terms of efficient attention design, the linear attention mechanism reduces the *O*(*N*^2^) computational complexity of standard self-attention to *O*(*N*) through kernel decomposition, significantly improving computational efficiency whilst maintaining global modeling capabilities. Su et al. (2024) explicitly encoded the relative spatial relationships by applying rotation transformations to the feature dimension, and widely validated as an effective position encoding strategy. Beyond visual inspection, attention mechanisms also demonstrate effectiveness in marine remote sensing tasks. For instance, Cai et al. (2025)integrate attention mechanisms into graph neural networks for SAR-based marine oil spill detection, achieving improved segmentation accuracy, suggesting that attention-based architectures hold promise across diverse ocean observation scenarios.

## 3 Methodology

### 3.1 Overview

We propose an improved YOLO object detection network for bridge defect detection. Based on the YOLOv10n architecture, the network incorporates three enhanced modules—the Partial Spatial Attention and Multi-layer Latent Linear Attention module (PSAMLLA), the Conditional Identity Block Feature Fusion module (C2fCIB), and the Dynamic Detection Head (DyHead)—to systematically optimize the original network across five dimensions: feature extraction, downsampling strategies, global context modeling, multi-scale feature fusion, and detection head adaptability. The overall network architecture is shown in Figure 1.

**Figure 1:**
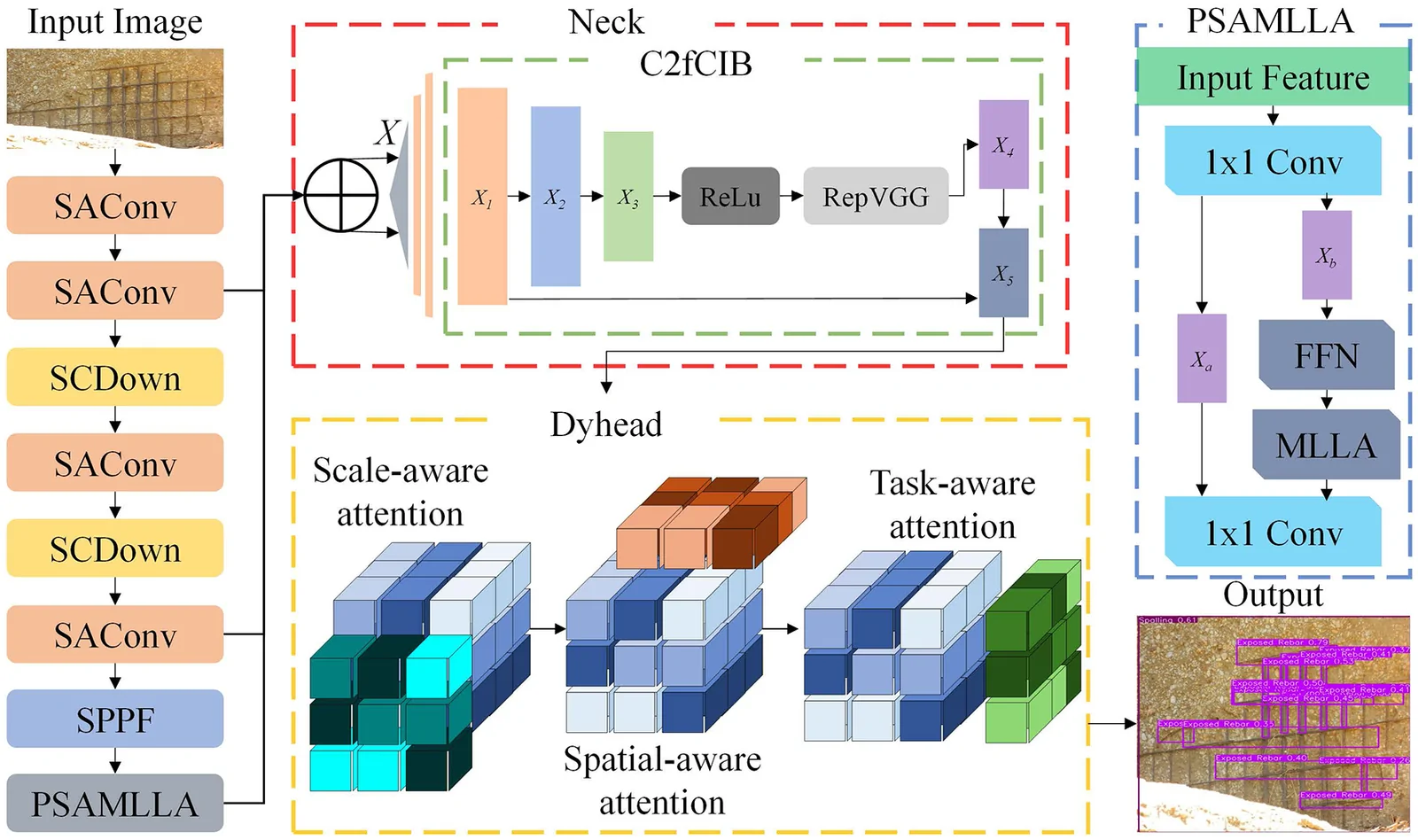
Overall network architecture.

The backbone network is responsible for extracting multi-level feature representations from the input images. For the input images of bridge defects, the backbone network progressively extracts multi-level features ranging from low-level textures to high-level semantics by alternately stacking convolutional layers, downsampling layers and feature transformation modules. Firstly, we employ Switchable Cavity Convolution (SAConv) as the internal feature extraction unit. By deploying *M* parallel 3 × 3 deep convolutional branches with varying cavity ratios, and combining this with an attention-weighted fusion strategy, we achieve adaptive adjustment of the receptive field. Subsequently, 1 × 1 convolutions are applied to learn from the input features, followed by Softmax normalization. This design enables the network to adaptively select the optimal receptive field based on the feature distribution of defects at different scales, effectively addressing the issue of insufficient multi-scale adaptability caused by the fixed receptive field of standard convolutions. Secondly, the two downsampling operations in the backbone network are replaced by the SCDown module, in place of standard strided convolutions. SCDown decouples channel transformation from spatial downsampling into two independent steps; simultaneously, the independent processing of each channel prevents the destruction of spatial structural information caused by cross-channel information aliasing. Finally, at the end of the backbone network, we introduce the PSAMLLA module to perform global context enhancement on the deepest-layer features. PSAMLLA employs a dual-branch partial attention design, performing linear attention computations on only half of the channels, thereby reducing the attention complexity from *O*(*N*^2^) to *O*(*N*) and providing higher-quality feature inputs for subsequent multi-scale feature fusion.

### 3.2 Backbone

The backbone network is responsible for the core task of extracting multi-level feature representations from input images. Given the characteristics of bridge defect detection scenarios—namely, significant variations in object scale, the dense distribution of small objects, and the substantial impact of defect texture details on detection accuracy—we have made two improvements to the YOLOv10n backbone network: firstly, we have replaced the standard Bottleneck structure in the original C2f module with a SAConv module to enable adaptive adjustment of the receptive field during feature extraction. The architecture of the SAConv module is shown in the Figure 2; secondly, by replacing the standard stride convolutions in the backbone network with the SCDown module, thereby decoupling channel transformation from spatial downsampling.

**Figure 2:**
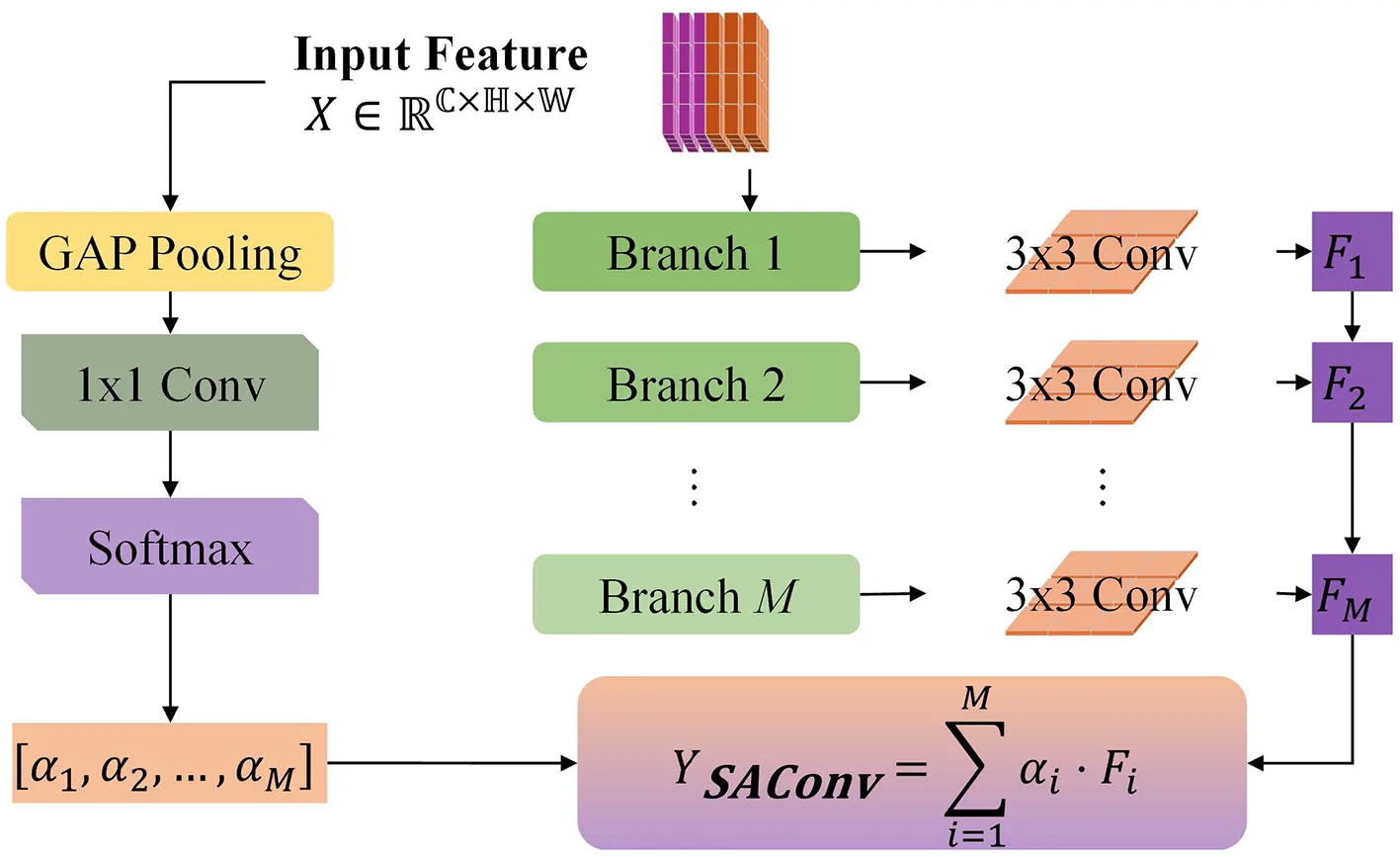
Architecture diagram of the SAConv module.

Standard convolutions have a fixed receptive field—a design choice that, while computationally efficient, fails to accommodate the extreme scale variations inherent in bridge defect detection, where fine cracks and large-area spalling must be recognized within the same feature extraction pathway. This limitation cannot be trivially resolved by simply enlarging the kernel size, as doing so would uniformly increase the receptive field and inevitably sacrifice local detail perception. Therefore, a dynamic, input-dependent receptive field adjustment mechanism is required—one that selectively expands the receptive field only when the input context indicates a large-scale defect, while preserving a narrow focus for crack-like patterns. In the case of bridge defects, small targets require a smaller receptive field to preserve local details, whilst large targets require a larger receptive field to capture global semantic information. To address this, we introduce the SAConv module. The core idea of SAConv is to enable the network to dynamically select the optimal receptive field based on the spatial distribution of the input features by deploying multiple convolutional branches with different dilation rates in parallel, combined with an attention-weighted fusion strategy. The SAConv module is illustrated in Figure 2. Specifically, SAConv deploys *M* parallel 3 × 3 convolutional branches with different dilation rates. For the input feature **X**∈ℝ^*C*×*H*×*W*^, the convolutional operation in the *i*th branch is:


$$\begin{array}{l} F_{i}=\text{BN}\left(\text{ReLU}\left(\text{Con}{\text{v}}_{3\times 3}^{d_{i}}\left(X\right)\right)\right),\;i=1,2,\ldots ,M \end{array}$$ (1)

Here, *d*_*i*_ denotes the sparsity of the *i*th branch. Convolutions with different sparsities have the same number of parameters, but their equivalent receptive fields differ, being 2*d*_*i*_+1 respectively. The outputs of each branch are fused via an attention-weighted summation. The attention weights are learned from the input features via a 1 × 1 convolution and normalized using Softmax:


$$\begin{array}{l} \left[\alpha_{1},\alpha_{2},\ldots ,\alpha_{M}\right]=\text{Softmax}\left(\text{Con}{\text{v}}_{1\times 1}\left(\text{GAP}\left(X\right)\right)\right) \end{array}$$ (2)

$$Y_{\text{SAConv}}=\sum_{i=1}^{M}\alpha_{i}\cdot F_{i}$$ (3)

We deploy SAConv in the form of C2f_SAConv within the intermediate layers of the backbone network, replacing the bottleneck structure in the standard C2f module. This enables the backbone network to adaptively adjust its receptive fields according to the scale features of different targets, thereby enhancing its ability to represent features of multi-scale defect targets.

Within the backbone network, downsampling operations are responsible for mapping high-resolution feature maps to lower resolutions, thereby progressively expanding the receptive field and enhancing the semantic abstraction capability of features. YOLOv10n originally employs 3 × 3 stride convolutions (stride = 2) for downsampling; this operation simultaneously achieves downsampling in the spatial dimension (halving the resolution) and mapping in the channel dimension (increased number of channels). However, this tightly coupled downsampling approach has the following shortcomings: firstly, standard convolution performs a cross-dimensional weighted summation of channels whilst downsampling spatially, which may lead to information aliasing between different channels, thereby interfering with the spatial structural information independently encoded by each channel; secondly, for bridge defect scenarios with a high density of small objects, standard stride convolution may lose key texture and edge information of small objects during the downsampling process.

To address these issues, we introduce the Spatial-Channel Downsampling module (SCDown), which decouples the downsampling process into two independent operations: channel transformation and spatial downsampling. Given an input feature $X\in {\mathbb{R}}^{C_{1}\times H\times W}$, SCDown first performs a mapping of the channel dimension via a 1 × 1 point convolution:


$$\begin{array}{l} X^{\prime }=\text{BN}\left(\text{ReLU}\left(\text{Con}{\text{v}}_{1\times 1}^{C_{2}}\left(X\right)\right)\right) \end{array}$$ (4)

Subsequently, a 3 × 3 deep convolution with a stride of *s* = 2 is applied to the channel-mapped features **X**′, and spatial downsampling is performed independently for each channel:


$$Y_{\text{SADown}}=BN\left({\text{DWConv}}_{3\times 3}^{s=2}\left(X^{\prime }\right)\right)$$ (5)

Final output features are $Y_{\text{SADown}}\in {\mathbb{R}}^{C_{2}\times H/2\times W/2}$.

SCDown offers two advantages over standard stride convolutions. In terms of parameter efficiency, standard 3 × 3 stride convolution requires *C*_1_×*C*_2_×9 parameters, whereas SCDown requires a total of *C*_1_×*C*_2_+*C*_2_×9 parameters. In terms of information preservation, SCDown's deep convolutions process each channel independently, thereby preventing the destruction of spatial structural information caused by cross-channel information aliasing. For small-scale defects such as bridge cracks and early-stage corrosion spots, their texture and edge information is typically concentrated in a few channels. SCDown's independent channel processing mechanism effectively preserves this critical information, preventing it from being diluted by cross-channel weighted operations during downsampling. By introducing SCDown at key resolution transition nodes within the backbone network, we have effectively improved the retention rate of spatial feature information during the downsampling process without significantly increasing computational overhead. This provides higher-quality multi-scale feature representations for subsequent feature pyramid fusion and object detection. It is worth noting that the core design rationale of SCDown is not parameter efficiency per se, but rather ensuring information fidelity through operation decoupling—a deeper design principle. The cross-channel aliasing caused by standard convolution's coupled weighted summation across channels during down-sampling is irreversibly destructive to micro-cracks and early-stage rust spots, whose texture information is concentrated in a small number of channels. SCDown fundamentally eliminates this information contamination by processing each channel independently via depthwise convolution.

### 3.3 Partial spatial attention and multi-layer latent linear attention modules

To enhance the spatial awareness of deep features in the backbone network, we introduce the PSAMLLA module. Its core idea is to utilize a linear attention mechanism in place of traditional self-attention, thereby significantly reducing computational complexity whilst maintaining global modeling capabilities. The PSAMLLA module employs a dual-branch partial attention design. Given an input feature **X**∈ℝ^*B*×*C*×*H*×*W*^, it is first mapped to a channel space of double the size via a 1 × 1 convolution, and then uniformly divided into two sub-features along the channel dimension **X**_*a*_ and **X**_*b*_:


$$\begin{array}{l} \left[X_{a},X_{b}\right]=\text{Split}\left(\text{Con}{\text{v}}_{1\times 1}\left(X\right),\frac{C^{\prime }}{2}\right) \end{array}$$ (6)

Here, *C*′ = *C*×*e* (where *e* = 0.5 is the channel scaling factor); **X**_*a*_, as the shortcut branch, retains the original feature information directly, whilst **X**_*b*_, as the attention branch, undergoes feature enhancement via a multi-head linear attention mechanism and a feedforward network. The computation process for the attention branch is as follows:


$$\begin{array}{l} X_{b}^{\prime }=X_{b}+\text{MLLA}\left(X_{b}\right) \end{array}$$ (7)

$$\begin{array}{l} X_{b}^{\prime \prime }=X_{b}^{\prime }+\text{FFN}\left(X_{b}^{\prime }\right) \end{array}$$ (8)

Here, MLLA stands for Multi-Layer Latent Attention, and FFN stands for Feedforward Network. Finally, the features from the two branches are concatenated along the channel dimension and then fused via a 1 × 1 convolution to produce the output:


$$\begin{array}{l} Y=\text{Con}{\text{v}}_{1\times 1}\left(\left[X_{a},X_{b}^{\prime \prime }\right]\right) \end{array}$$ (9)

It is worth emphasizing in deep visual features, the attention distributions across different channels tend to saturate and homogenize, meaning that not all channels require expensive global attention modeling. Saturation—excessive attention computation not only introduces redundant overhead but may also inject noise, while the shortcut branch serves as a feature protection mechanism that effectively prevents detail loss caused by over-attention.

The core component of PSAMLLA, MLLAttention, is an efficient linear attention mechanism. Its architecture is shown in Figure 3. First, the input features $X\in {\mathbb{R}}^{B\times C^{\prime }\times H\times W}$ are reshaped into a sequence **X**∈ℝ^*B*×*N*×*C*^′, where *N* = *H*×*W* is the sequence length. The query **Q** and key **K** are obtained via linear projection, with the input features themselves serving as the value **V**:


$$\begin{array}{l} \left[Q,K\right]=\text{Linear}\left(X\right) \end{array}$$ (10)

$$\begin{array}{l} V=X \end{array}$$ (11)

Unlike the standard attention mechanism, which has a computational complexity of *O*(*N*^2^), MLLAttention ensures strict non-negativity by applying an ELU activation function to both the query and the key, and adding a bias:


$$\begin{array}{l} Q^{\prime }=\text{ELU}\left(Q\right)+1 \end{array}$$ (12)

$$\begin{array}{l} K^{\prime }=\text{ELU}\left(K\right)+1 \end{array}$$ (13)

Here, **1** denotes a tensor of all ones with the same shape as the corresponding matrix. Based on the non-negativity constraint, the attention computation can be decomposed into a cascaded form consisting of key-value matrix multiplication and query matrix multiplication. Additionally, rotational position encoding is introduced for **Q**′ and **K**′ to enhance spatial position awareness:


$$\begin{array}{l} \hat{Q}=\Phi \left(Q^{\prime }\right) \end{array}$$ (14)

$$\begin{array}{l} \hat{K}=\Phi \left(K^{\prime }\right) \end{array}$$ (15)

Here, Φ(·) denotes the rotational position encoding operator. The normalization factor **Z** is computed as the product of the query matrix and the mean vector of the keys:


$$\begin{array}{l} Z=\frac{1}{Q^{\prime }\cdot {\bar{K^{\prime }}}^{⊤}+\varepsilon } \end{array}$$ (16)

**Figure 3:**
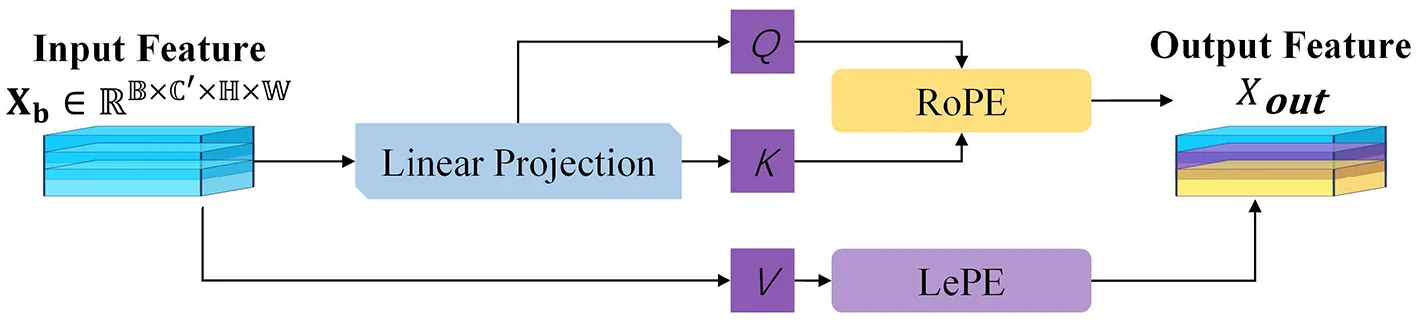
Architecture diagram of the MLLAttention.

Here, $\bar{K^{\prime }}=\frac{1}{d_{h}}\overset{d_{h}}{\underset{i=1}{\sum }}K_{i}^{\prime }$ represents the mean of the keys across the feature dimensions, and ε is the numerical stability constant. The final linear attention output is computed via key-value aggregation and query weighting:


$$\begin{array}{l} X_{\text{attn}}=\hat{Q}\cdot \left(\frac{{\hat{K}}^{⊤}}{\sqrt{N}}\cdot \frac{V}{\sqrt{N}}\right)⊙Z \end{array}$$ (17)

The above calculation reduces the *O*(*N*^2^) complexity of standard self-attention to *O*(*N*), significantly improving computational efficiency. Furthermore, the attention output is combined with the output of learnable position encoding (LePE):


$$\begin{array}{l} X_{\text{out}}=X_{\text{attn}}+\text{LePE}\left(V\right) \end{array}$$ (18)

In particular, LePE models local positional bias via a 3 × 3 deep convolutional layer DWConv_3 × 3_, effectively addressing the shortcomings of linear attention in terms of local feature perception. As shown in Figure 4.

**Figure 4:**
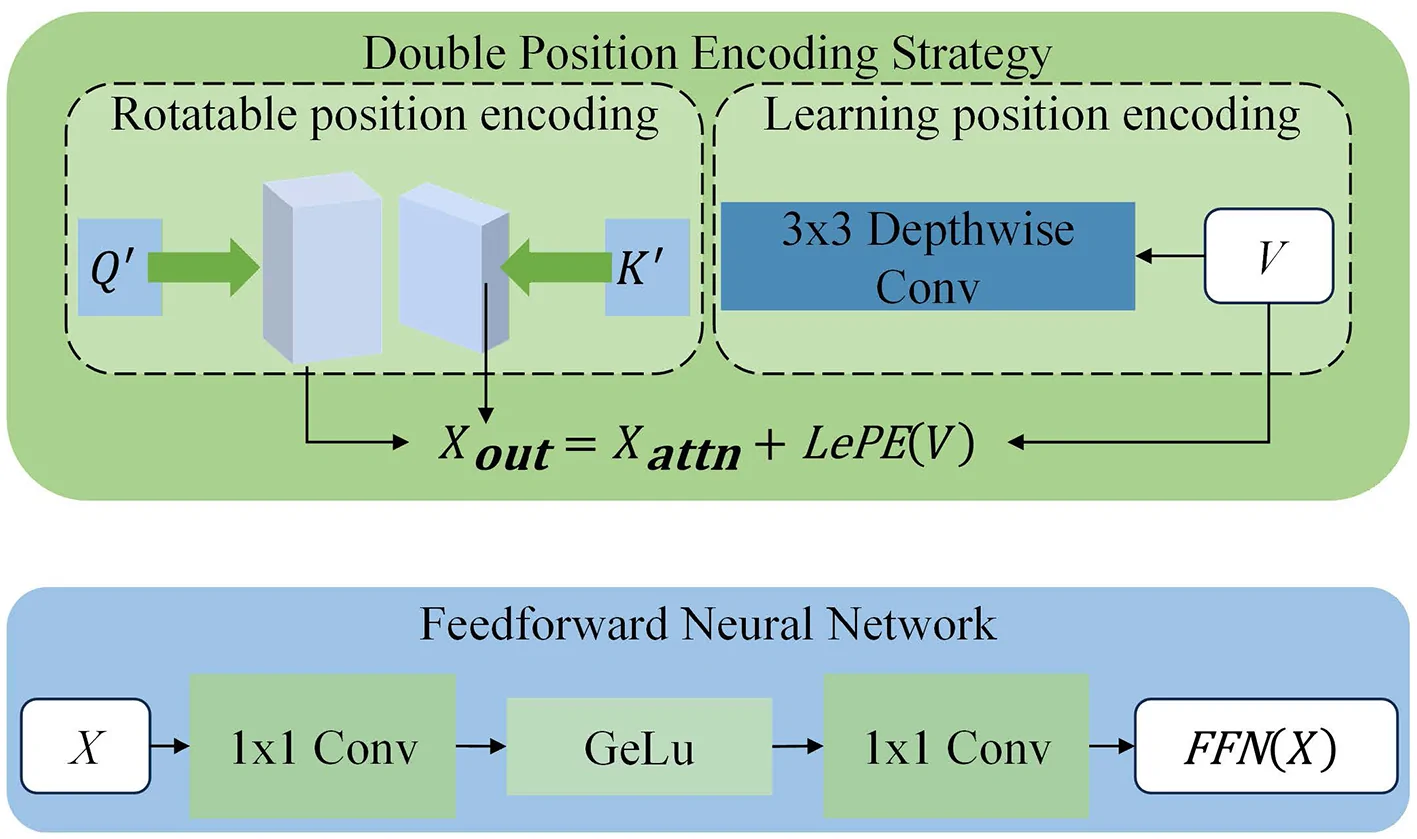
Architecture diagram of the RoPE and LePE.

Rotational Position Encoding (RoPE) explicitly encodes the relative relationships between spatial positions by grouping feature dimensions in pairs, treating them as complex numbers and applying a rotation transformation. This is illustrated in Figure 4. For a spatial position (*i, j*) and a feature dimension pair (2*k*, 2*k*+1), the rotation angle is defined as:


$$\begin{array}{l} \theta_{i,j}^{\left(k\right)}=\frac{i+j\cdot H}{\beta^{2k/d}} \end{array}$$ (19)

Here, β is the fundamental frequency, *d* is the feature dimension, and *H* is the height of the feature map. The rotation operation is equivalent to multiplication in the complex number field:


$$\begin{array}{l} \Phi {\left(x\right)}_{2k}=x_{2k}\cos\left(\theta_{i,j}^{\left(k\right)}\right)-x_{2k+1}\sin\left(\theta_{i,j}^{\left(k\right)}\right) \end{array}$$ (20)

$$\begin{array}{l} \Phi {\left(x\right)}_{2k+1}=x_{2k}\sin\left(\theta_{i,j}^{\left(k\right)}\right)+x_{2k+1}\cos\left(\theta_{i,j}^{\left(k\right)}\right) \end{array}$$ (21)

This rotation encoder inherently possesses relative position sensing capabilities; when the relative positional offset between a query and a key is (Δ*i*, Δ*j*), their dot product depends solely on the relative displacement, thereby enhancing the model's ability to capture global spatial relationships.

The feedforward network within the attention branch consists of two 1 × 1 convolutions and employs an expand-contract bottleneck structure:


$$\begin{array}{l} FFN\left(X\right)=\text{Con}{\text{v}}_{1\times 1}^{2C^{\prime }}\left(\text{GELU}\left(\text{Con}{\text{v}}_{1\times 1}^{C^{\prime }}\left(X\right)\right)\right) \end{array}$$ (22)

In this configuration, the number of channels in the hidden layer is expanded to 2*C*′, with the GELU activation function applied; no activation function is used in the output layer. The output of the feedforward network is added to the input via a residual connection, ensuring the stability of gradient propagation in deep networks.

We deploy the PSAMLLA module after the SPPF module and before the Feature Pyramid Network (FPN) Neck in the YOLO backbone network to perform spatial attention enhancement on the deepest features output by the backbone network. This module efficiently captures global contextual information through a linear attention mechanism, supplemented by a dual-position encoding strategy that balances local and global spatial awareness. At the same time, the partial attention design keeps computational complexity to a minimum, enabling the model to focus more precisely on target regions and enhancing the detection head's localization accuracy and classification capabilities for multi-scale targets.

### 3.4 Conditional identity block feature fusion module

In the cross-scale fusion path of the feature pyramid, feature maps at different scales contain rich multi-granularity information after being concatenated; however, direct fusion via standard convolution struggles to fully capture spatial dependencies within the channels. To address this, we introduce the Conditional Identity Block Feature Fusion module (C2fCIB), an improvement upon the feature fusion design of YOLOv9. This module replaces the bottleneck structure in the standard C2f module with conditional identity blocks as the basic computational unit, thereby enhancing the expressive power of feature fusion whilst maintaining a lightweight architecture.

CIB adopts a bottleneck structure design with full-depth convolutions. Given an input feature **X**∈ℝ^*C*×*H*×*W*^, the computational process of CIB can be expressed as:


$$\begin{array}{l} X_{1}=\text{BN}\left(\text{ReLU}\left(\text{DWCon}{\text{v}}_{3\times 3}\left(X\right)\right)\right) \end{array}$$ (23)

$$\begin{array}{l} X_{2}=\text{BN}\left(\text{ReLU}\left(\text{Con}{\text{v}}_{1\times 1}^{2C^{\prime }}\left(X_{1}\right)\right)\right) \end{array}$$ (24)

$$\begin{array}{l} X_{3}=\text{RepVGGDW}\left(X_{2}\right) \end{array}$$ (25)

$$\begin{array}{l} X_{4}=\text{BN}\left(\text{ReLU}\left(\text{Con}{\text{v}}_{1\times 1}^{C}\left(X_{3}\right)\right)\right) \end{array}$$ (26)

$$\begin{array}{l} X_{5}=\text{BN}\left(\text{DWCon}{\text{v}}_{3\times 3}\left(X_{4}\right)\right) \end{array}$$ (27)

$$Y_{\text{CIB}}=X+X_{5}$$ (28)

Here, *C*′ = *C*×*e* represents the number of channels in the hidden layer (where *e* is the expansion ratio), DWConv denotes deep convolution, and RepVGGDW denotes a RepVGG-style deep convolution module with large kernels.

The RepVGGDW module draws on the design philosophy of the RepVGG network, introducing a multi-branch training strategy based on deep convolutions. During the training phase, this module executes three branches in parallel: 3 × 3 deep convolutions, 1 × 1 deep convolutions, and an identity mapping, and fuses the outputs via element-wise summation:


$$\begin{array}{l} Y_{\text{RepVGGDW}}=\text{DWCon}{\text{v}}_{3\times 3}\left(X\right)+\text{DWCon}{\text{v}}_{1\times 1}\left(X\right)+X \end{array}$$ (29)

During the inference stage, structural reparameterization is used to equivalently transform the three branches described above into a single 3 × 3 deep convolution, thereby enabling large-scale feature extraction with zero additional computational overhead. The C2fCIB module inherits the overall framework of C2f, replacing the bottleneck with a CIB as the internal feature transformation unit. For the input feature $X\in {\mathbb{R}}^{C_{1}\times H\times W}$, it is first divided into two parts via a 1 × 1 convolution: one part serves directly as the inter-layer connection feature, whilst the other undergoes deep feature transformation through *N* stacked CIB modules. Subsequently, the two feature parts are concatenated along the channel dimension and then fused via a 1 × 1 convolution to produce the output:


$$Y_{\text{C2fCIB}}={\text{Conv}}_{1\times 1}\left(\text{Concat}\left(X_{\text{shortcut}},{\text{CIB}}^{N}\left(X_{\text{transform}}\right)\right)\right)$$ (30)

Here, *N* denotes the number of stacked CIB modules. We deploy the C2fCIB module on the deepest fusion path of the feature pyramid neck, where it receives the concatenated results of deep and intermediate features from the backbone network. Through a lightweight design utilizing full-depth convolutions, this module reduces the number of parameters and computational overhead. Meanwhile, the deep convolutions of the RepVGG core effectively expand the receptive field, enabling the fused features to capture both local details and global semantic information simultaneously. This provides high-quality feature inputs for the precise localization and classification performed by the detection head.

### 3.5 Dynamic detection head

Traditional object detectors typically process feature maps at different scales within the feature pyramid using separate detection heads; this design overlooks the intrinsic correlations between features at different scales. Furthermore, there are fundamental differences in the requirements for features between classification and regression tasks: classification tasks focus more on semantic information, whilst regression tasks focus more on spatial localization accuracy. To address these issues, we introduce a dynamic detection head, which unifies scale-aware, spatially-aware and task-aware processing through deformable convolutions and a dynamic attention mechanism.

The core computational unit of the dynamic detection head is the modulated deformable convolution. For an input feature **X**∈ℝ^*C*×*H*×*W*^, modulated deformable convolution dynamically adjusts the sampling position and feature response intensity by predicting an offset Δ**p** and a modulation scalar **m**. The output of the convolution kernel at position **p**_0_ is computed as:


$$\begin{array}{l} Y\left(p_{0}\right)=\overset{K}{\underset{k=1}{\sum }}m_{k}\cdot W_{k}\cdot X\left(p_{0}+\Delta p_{k}\right) \end{array}$$ (31)

Here, *K* = *k*×*k* denotes the number of sampling points covered by the convolution kernel, **W**_*k*_ denotes the convolution kernel weights corresponding to the *k*th sampling position, and Δ**p**_*k*_ and **m**_*k*_ are learned from the input features via the offset prediction branch and the modulation mask prediction branch, respectively:


$$\begin{array}{l} \left[\Delta p,\hat{m}\right]=f_{\text{offset}}\left(X\right) \end{array}$$ (32)

$$\begin{array}{l} m=\sigma \left(\hat{m}\right) \end{array}$$ (33)

Here, σ(·) denotes the sigmoid activation function, which constrains the modulation scalar to the interval [0, 1].

We have designed three complementary attention mechanisms that realize scale-aware, spatial-aware and task-aware attention, respectively, as shown in Figure 5. For the feature **F**_*l*_ at layer *l* of the feature pyramid, scale-aware attention enables multi-scale feature interaction through cross-scale deformable convolutions. Specifically, three deformable convolution branches are applied to **F**_*l*_:


$$\begin{array}{l} T_{l}^{\left(1\right)}=f_{\text{DyConv}}^{\left(1\right)}\left(F_{l};\Delta p,m\right) \end{array}$$ (34)

$$\begin{array}{l} T_{l}^{\left(2\right)}=f_{\text{DyConv}}^{\left(2\right)}\left(F_{l-1};\Delta p,m\right) \end{array}$$ (35)

$$\begin{array}{l} T_{l}^{\left(3\right)}=\text{Resize}\left(f_{\text{DyConv}}^{\left(3\right)}\left(F_{l+1};\Delta p,m\right)\right) \end{array}$$ (36)

In particular, $f_{\text{DyConv}}^{\left(1\right)}$ operates at the current scale, $f_{\text{DyConv}}^{\left(2\right)}$ operates on adjacent lower-level features (cross-scale downsampling), $f_{\text{DyConv}}^{\left(3\right)}$ operates on adjacent higher-level features (cross-scale upsampling), and Resize(·) denotes a bilinear interpolation alignment operation. The three branches share the same set of offsets and modulation masks, enabling cross-scale information exchange.

**Figure 5:**
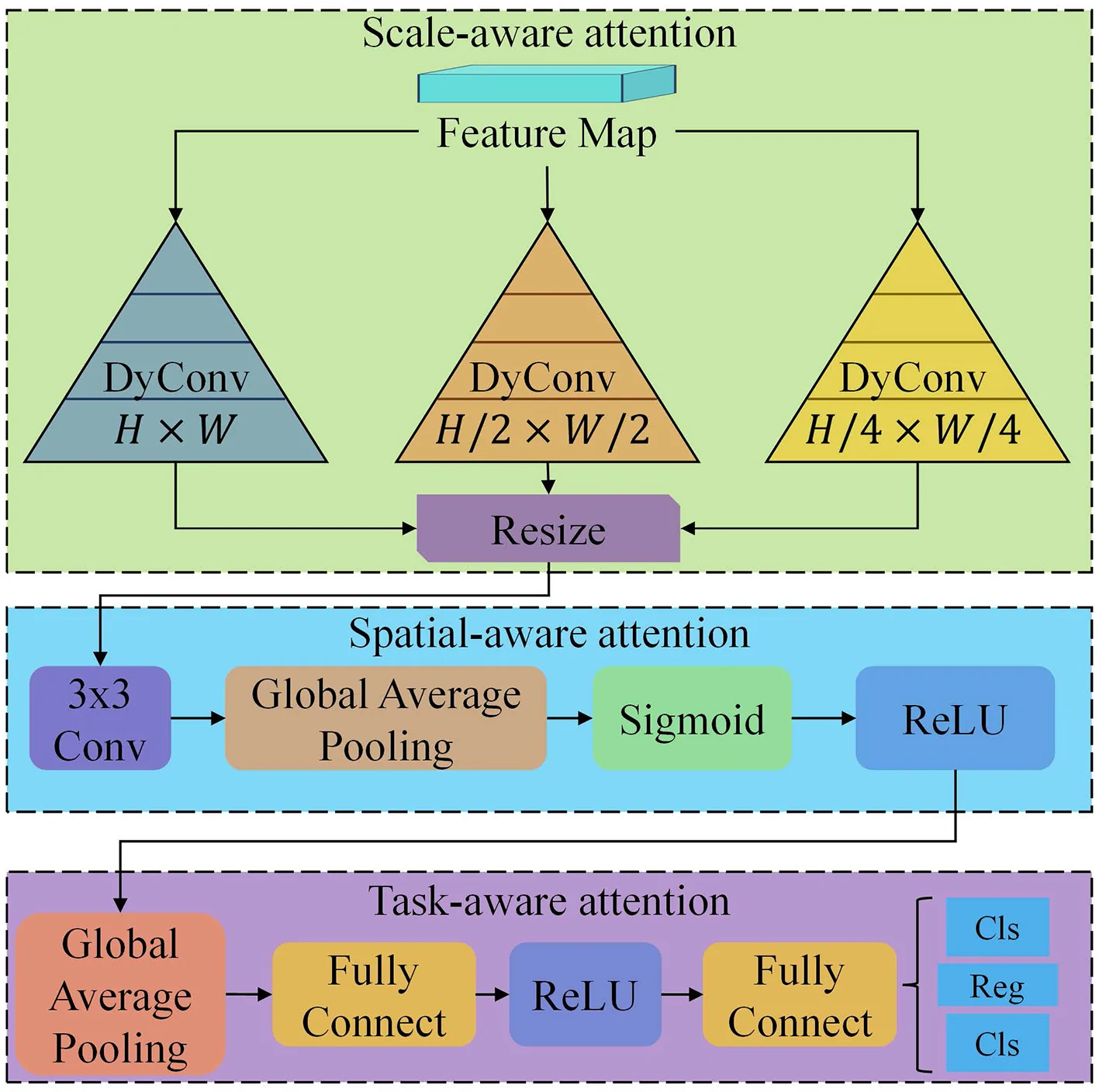
Architectural diagram of the triple attention mechanism.

Spatial-aware attention generates spatial attention weights via global average pooling and a Sigmoid activation function, which are used to weight feature responses from different locations:


$$\begin{array}{l} S_{l}=h_{\sigma }\left(\text{GAP}\left(T_{l}\right)\right) \end{array}$$ (37)

Here, GAP(·) denotes the global average pooling operation, and *h*_σ_(·) denotes an attention function composed of a 1 × 1 convolution and an *h*-Sigmoid activation function:


$$\begin{array}{l} h_{\sigma }\left(x\right)=\frac{\text{ReL}{\text{U}}_{6}\left(x+3\right)}{6}\cdot H_{\max} \end{array}$$ (38)

Here, *H*_max_ denotes the maximum scaling factor. The weighted aggregation of multi-scale fusion features is achieved through element-wise multiplication by attention weights:


$$\begin{array}{l} {\hat{F}}_{l}=\frac{1}{K_{s}}\overset{K_{s}}{\underset{k=1}{\sum }}T_{l}^{\left(k\right)}⊙S_{l}^{\left(k\right)} \end{array}$$ (39)

Here, *K*_*s*_ denotes the number of feature branches involved in aggregation.

Task-aware attention achieves differentiated feature representations for classification and regression tasks through a dynamic ReLU activation function. The dynamic ReLU learns independent activation function parameters for each channel:


$$\begin{array}{l} \text{DyReLU}\left(x\right)=\max\left(\overset{\left(c\right)}{\underset{1}{a}}\cdot x_{c}+b_{1}^{\left(c\right)},a_{2}^{\left(c\right)}\cdot x_{c}+b_{2}^{\left(c\right)}\right) \end{array}$$ (40)

Here, *c* denotes the channel index, and the activation parameters $\left\{a_{1}^{\left(c\right)},b_{1}^{\left(c\right)},a_{2}^{\left(c\right)},b_{2}^{\left(c\right)}\right\}$ are dynamically generated from the input features via global average pooling and two layers of fully connected networks:


$$\begin{array}{l} \left\{a_{1},b_{1},a_{2},b_{2}\right\}=h_{\sigma }\left(\text{F}{\text{C}}_{2}\left(\text{ReLU}\left(\text{F}{\text{C}}_{1}\left(\text{GAP}\left({\hat{F}}_{l}\right)\right)\right)\right)\right) \end{array}$$ (41)

The dual-branch maximum-value design provides the activation function with a more flexible non-linear representation, enabling different channels to adopt distinct activation strategies according to task requirements.

The overall architecture of the Dynamic Detection Head consists of three stacked DyConv modules, corresponding to the three scales of the feature pyramid. Each DyConv module sequentially performs three types of attention operations: scale-aware, spatial-aware, and task-aware. The output features from the DyConv tower are then fed into the classification and regression branches:


$$\begin{array}{l} P_{\text{reg}}^{\left(l\right)}=f_{\text{reg}}\left({\hat{F}}_{l}\right) \end{array}$$ (42)

$$\begin{array}{l} P_{\text{cls}}^{\left(l\right)}=f_{\text{cls}}\left({\hat{F}}_{l}\right) \end{array}$$ (43)

Here, *f*_reg_ and *f*_cls_ denote the regression branch and the classification branch, respectively, each consisting of two 3 × 3 convolutions and one 1 × 1 convolution. The regression branch outputs a distribution representation with 4 × *R*_max_ channels, which is integrated using DFL (Distribution Focal Loss) to obtain the final bounding box coordinates; the classification branch outputs predicted probabilities for *N*_*c*_ classes.

We deploy the dynamic detection head after the YOLO feature pyramid's Neck, replacing the original detect head. This detect head utilizes deformable convolutions to achieve cross-scale feature interaction and adaptive spatial sampling, and employs dynamic ReLU to implement differentiated feature activation for classification and regression tasks. Without significantly increasing the number of parameters or computational cost, it effectively enhances the detection performance of the model for multi-scale, multi-class objects.

## 4 Experiments

Our experiments were conducted on a system with an NVIDIA GeForce RTX 4080 (16GB VRAM) and an Intel Core i5-13600KF processor, as detailed in Table 1. The software environment is based on the PyTorch 2.0.1 deep learning framework and the CUDA 11.8 acceleration library.

**Table 1:** Experimental environment configuration.

Component	Specification/version
Processor (CPU)	Intel(R) Core(TM) i5-13600KF @ 3.50 GHz
Graphics Card (GPU)	NVIDIA GeForce RTX 4080
Memory (RAM)	16 GB
Operating System (OS)	Windows 10 Professional (64-bit)
Programming Language	Python 3.9.18
Deep Learning Framework	PyTorch 2.0.1
Acceleration Library	CUDA 11.8/cuDNN

The input image dimensions were uniformly resized to 640 × 640, the batch size was set to 32, and the total number of training epochs was set to 500. The initial learning rate was set to 0.01 and decayed using a cosine annealing strategy, as shown in Table 2.

**Table 2:** Experimental model configuration parameters.

Parameter name	Setting value
Input image size	640 × 640
Model iterations (Epochs)	500
Batch size	32
Optimizer	SGD
Initial learning rate	0.01
Final learning rate	0.0001
Learning rate momentum	0.937
Weight decay coefficient	0.0005
Warmup iterations	3
Warmup momentum	0.8
Warmup initial learning rate	0.1

### 4.1 Statistical analysis of the dataset

The bridge defect dataset used in this study was sourced from the publicly available “bridge-bkqxz/2” dataset on the Roboflow Universe platform. Upon export, the dataset was randomly divided into three independent subsets according to the platform's default configuration: a training set (70%), a validation set (15%), and a test set (15%). Specifically, the training set is used for model parameter learning and optimization; the validation set is employed for hyperparameter tuning, model selection, and intermediate performance monitoring during training; the test set is strictly held out and used exclusively for final generalization performance evaluation after the entire training process is completed, and it has never participated in any training or tuning procedures throughout the experiments. This split ratio adheres to the standard export settings of the Roboflow platform and is also consistent with common practices in the object detection community.

The bridge defect dataset we used comprises 20 defect categories. It is sourced from the publicly available dataset bridge-bkqxz/2 on the RoboflowUniverse platform, released under the CC BY 4.0 open-source license, and covers a range of typical bridge defects, including cracks, efflorescence, deep cracks and concrete spalling. To ensure the validity of the data set and the robustness of the training strategy, we conducted a systematic statistical analysis of the data set in three dimensions: class distribution, spatial distribution of the bounding boxes, and distribution of target scales.

During training, we employed the official default data augmentation strategies of YOLOv10. Specifically, Mosaic augmentation is applied for the first 250 epochs by concatenating four randomly scaled and cropped images to enhance multi-scale and small-object perception, and is disabled thereafter. MixUp augmentation is randomly enabled with a probability of 0.5 throughout training to improve generalization and noise robustness. Random horizontal flipping is applied with a probability of 0.5 to increase spatial diversity. Additionally, random scaling and cropping are applied during Mosaic composition to simulate scale variations. All augmentation strategies are applied exclusively to the training set; the validation and test sets undergo only resize and normalization without any augmentation, ensuring unbiased evaluation.

The bar chart in the top-left of Figure 6 shows the instance count per class. Experimental results indicate that the dataset exhibits significant class imbalance. Among these, the Crack class dominates with 15,952 instances, whilst the Efflorescence class ranks second with 11,385 instances. In contrast, a few categories, such as Faunal Biodegradation and Corrosion, contain only a little over a hundred instances, with the difference in instance counts between the largest and smallest categories exceeding two orders of magnitude. This long-tail distribution poses a severe challenge to model training: during training, the model tends to overfit high-frequency categories whilst neglecting the learning of low-frequency categories, directly leading to a significant decline in detection accuracy for the minority categories.

**Figure 6:**
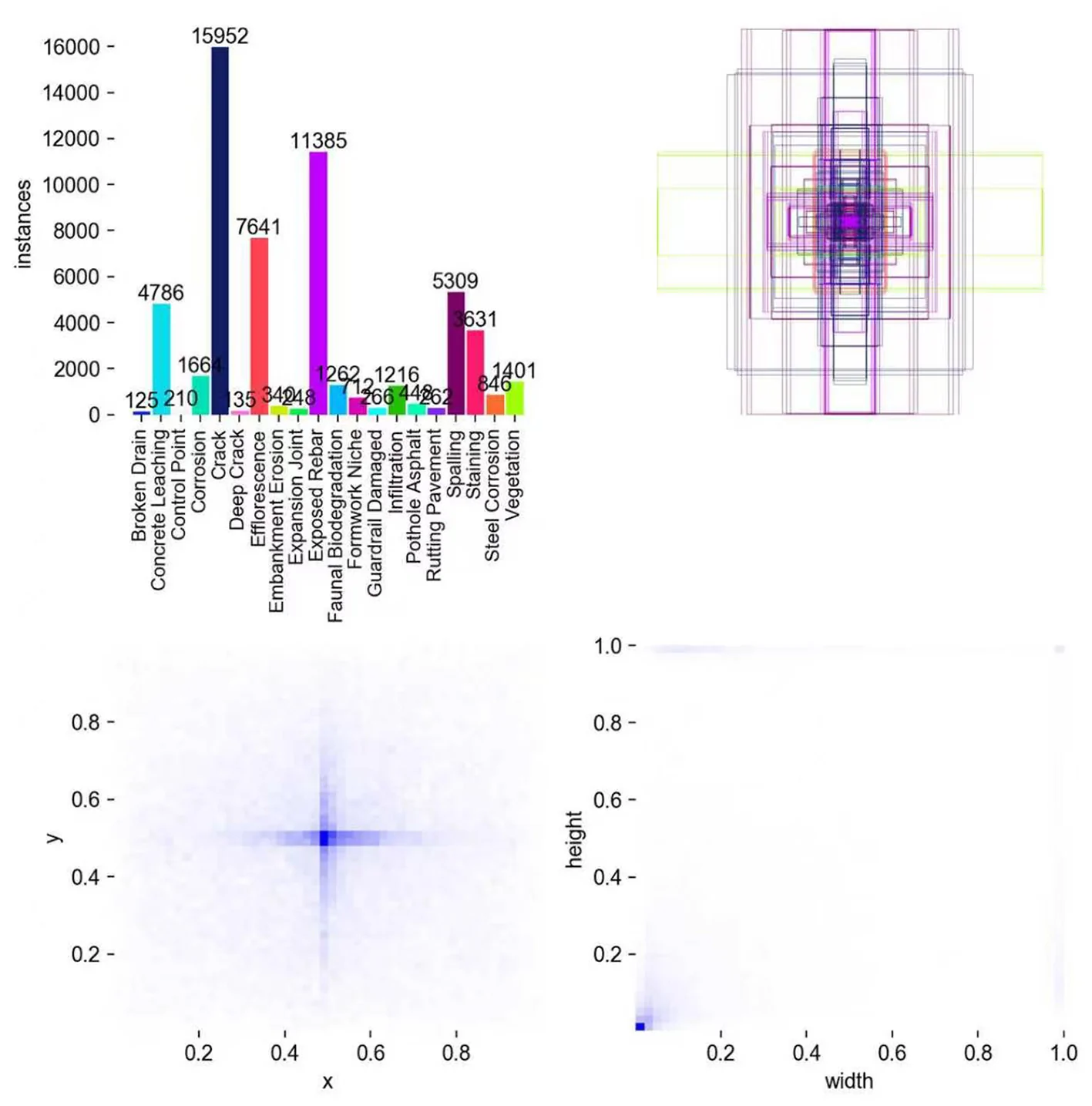
Statistical analysis of the dataset.

The bounding box overlay visualization in the top-right corner of Figure 6, together with the heatmap of bounding box center coordinates in the bottom-left corner, jointly reveal the spatial distribution patterns of the targets. Statistical results indicate that the centers of the bounding boxes for a large number of defect targets are concentrated in the central region of the image, exhibiting a distinct cross-shaped high-density distribution. This distribution characteristic is closely related to the method of acquiring bridge inspection images: when capturing images manually, operators typically position the defect area at the center of the frame to obtain the optimal viewing angle. This spatial prior information provides an important reference for the subsequent design of the detection head.

The heatmap of bounding box width and height distributions in the bottom-right corner of Figure 6 illustrates the statistical scale characteristics of the targets. Analysis reveals that the normalized width and height of the vast majority of defect targets are both less than 0.2, exhibiting a typical distribution dominated by small targets. Under the evaluation criteria of the COCO dataset, targets with a normalized area smaller than 32 × 32 pixels are defined as small targets. Bridge defects (such as micro-cracks and early-stage corrosion spots) typically occupy only a very small visual area within the entire image. Coupled with factors such as the considerable shooting distance at the site and uneven ambient lighting, this makes small-object detection one of the core challenges of this task.

### 4.2 Ablation experiment

To validate the effectiveness of the proposed modules and their contribution to bridge defect detection performance, we conducted ablation experiments by progressively adding modules. The experiments used the original YOLOv10-n model as the baseline, sequentially introducing backbone improvements (Backbone: SAConv and SCDown), the PSAMLLA partial self-attention multi-level attention module, the C2fCIB context information bottleneck module, and the DyHead dynamic detection head, ultimately forming the complete improved model. The experimental results are shown in Table 3.

**Table 3:** Comparison of experimental results across all categories for different module combinations. The Backbone denotes SAConv and SCDown.

Module	Precision	Recall	mAP_50_	mAP_50-95_
Baseline	0.327	0.269	0.245	0.168
Baseline+SAConv	0.331	0.278	0.247	0.171
Baseline+SCDown	0.330	0.275	0.246	0.169
Baseline+Backbone	0.334	0.285	0.247	0.172
Baseline+Backbone+PSAMLLA	0.335	0.291	0.253	0.179
Baseline+Backbone+PSAMLLA+C2fCIB	0.341	0.297	0.256	0.182
Full Model	0.346	0.301	0.259	0.184

As can be seen from Table 3, each improvement module demonstrates consistent positive gains across all evaluation metrics, with no instances of negative transfer observed. This indicates that the design of each module is sound and that they are well-compatible with one another. Compared with the Baseline model, the fully enhanced model achieved improvements of 5.81%, 11.52%, 5.71%, and 9.52% respectively, with the improvement in recall being the most significant. In the task of bridge defect detection, the structural safety risks potentially caused by missed detections far outweigh the additional manual review costs resulting from false positives; therefore, the substantial improvement in recall holds significant value for engineering applications.

During the backbone network refinement phase, the introduction of the SAConv module—which employs switchable dilated convolutions—alongside the use of the SCDown module to replace standard stride convolutions for downsampling, resulted in a 1.6% increase in recall rate, making it the most significant contributor among the four modules. These results indicate that by adaptively adjusting the dilation rate, SAConv enables the model to dynamically capture multi-scale lesion features without significantly increasing computational overhead, thereby effectively enhancing detection coverage for distant small targets and low-contrast lesions; meanwhile, the SCDown module mitigates the loss of fine-grained defect information during feature dimensionality reduction through a more refined downsampling strategy. It is worth noting that the improvements to the backbone network yield a relatively limited increase in mAP_50_; this may be attributed to the multi-branch structure of SAConv not having fully converged under the current training settings, meaning that the enhancement in its semantic discrimination capabilities has not been fully realized within a short timeframe.

Following the introduction of the PSAMLLA module at the end of the backbone network, mAP_50_ and mAP_50-95_ improved by 0.006 and 0.007 respectively, with the improvement in mAP_50-95_ ranking first among all single modules. Using a partial self-attention mechanism to facilitate cross-level information exchange across both channel and spatial dimensions, PSAMLLA significantly improves the model's ability to precisely localize features within the boundaries of the lesions. The fact that mAP_50-95_ shows a greater improvement compared to mAP_50_ indicates that PSAMLLA not only helps the model detect defect targets more effectively, but also significantly improves the quality of overlap between predicted bounding boxes and ground-truth annotations. This is of critical importance for the quantitative assessment and severity determination of bridge defects.

Following the introduction of the C2fCIB module, both precision and recall improved by 0.006, demonstrating a balanced increase in both metrics. Through its compression-reconstruction feature processing workflow, C2fCIB effectively filters out background interference such as bridge surface textures, water stains and shadows, thereby enhancing the purity of feature representation in defect areas. The simultaneous improvement in precision and recall indicates that this module plays a positive role in both reducing false positives (suppressing background noise) and reducing false negatives (preserving weak defect features).

Finally, the introduction of the DyHead dynamic detection head at the detection stage achieves joint optimisation of classification and localization through a triple attention mechanism comprising scale-aware, spatial-aware and task-aware components. This further increases precision by 0.005 to 0.346, with the comprehensive performance of all metrics reaching an optimal level. Taking the above analysis into account, the improvement rate of mAP_50-95_ (9.52%) exceeds that of mAP_50_ (5.71%), indicating that our method not only effectively increases the detection rate of defects but also significantly improves the localization accuracy of detection boxes. This characteristic is particularly important for bridge defect detection tasks, as precise boundary localization is a fundamental prerequisite for quantifying defect dimensions, automatically grading severity, and tracking deterioration trends, thereby providing more reliable technical support for the intelligent assessment of bridge structural health.

### 4.3 Analysis of experimental results

To further evaluate the improved model's detection capabilities across different types of bridge defects, we calculated the precision, recall, mAP_50_ and mAP_50-95_ for each category on the validation and test sets, respectively. The results are shown in Tables 4, 5.

**Table 4:** Performance metrics for our method by category on the validation set.

Defect class	Precision	Recall	mAP_50_	mAP_50-95_
All	0.349	0.304	0.268	0.189
Broken drain	0.329	0.167	0.14	0.108
Concrete leaching	0.294	0.26	0.2	0.119
Control point	0.906	1	0.995	0.984
Corrosion	0.12	0.0554	0.0587	0.0322
Crack	0.672	0.645	0.67	0.51
Deep crack	0.125	0.036	0.0618	0.0395
Efflorescence	0.306	0.288	0.219	0.124
Embankment erosion	0.325	0.495	0.313	0.179
Expansion joint	0.284	0.398	0.31	0.212
Exposed rebar	0.423	0.31	0.282	0.161
Faunal biodegradation	0.301	0.25	0.211	0.148
Formwork niche	0.192	0.0663	0.0565	0.0272
Guardrail damaged	0.28	0.328	0.236	0.113
Infiltration	0.374	0.191	0.177	0.101
Pothole asphalt	0.354	0.511	0.345	0.213
Rutting pavement	0.263	0.173	0.204	0.0919
Spalling	0.483	0.286	0.305	0.223
Staining	0.316	0.196	0.191	0.11
Steel corrosion	0.256	0.116	0.102	0.0589
Vegetation	0.372	0.308	0.276	0.222

**Table 5:** Performance metrics for our method by category on the test set.

Defect class	Precision	Recall	mAP_50_	mAP_50-95_
All	0.346	0.301	0.259	0.184
Broken drain	0.338	0.11	0.187	0.121
Concrete leaching	0.358	0.31	0.269	0.159
Control point	0.923	1	0.995	0.949
Corrosion	0.106	0.08	0.0491	0.0254
Crack	0.637	0.591	0.599	0.454
Deep crack	0.201	0.0673	0.0897	0.05
Efflorescence	0.343	0.321	0.255	0.151
Embankment erosion	0.247	0.576	0.245	0.158
Expansion joint	0.347	0.2	0.185	0.106
Exposed rebar	0.42	0.334	0.304	0.165
Faunal biodegradation	0.301	0.195	0.165	0.112
Formwork niche	0.225	0.0843	0.0734	0.0296
Guardrail damaged	0.22	0.346	0.218	0.162
Infiltration	0.401	0.273	0.226	0.15
Pothole asphalt	0.218	0.341	0.225	0.12
Rutting pavement	0.188	0.214	0.187	0.127
Spalling	0.538	0.364	0.373	0.281
Staining	0.3	0.191	0.184	0.109
Steel corrosion	0.383	0.275	0.258	0.171
Vegetation	0.234	0.127	0.0909	0.0702

In terms of overall metrics, our method achieved mAP_50_ and mAP_50-95_ on the test set are 0.259 and 0.184 respectively, representing a decrease of only 0.009 and 0.005 compared to the validation set. This indicates that the model maintains good generalization performance on unseen data, with no significant overfitting observed.

Across all categories, the Control Point (control point label) achieved near-perfect detection performance, with mAP_50_ reaching 0.995 on both the validation and test sets, a precision exceeding 0.90, and a recall of 1.000. As a reference label manually set during the dataset annotation process, the Control Point possesses highly consistent geometric shapes, colors and dimensions. Its outstanding performance validates the effectiveness of the model's fundamental detection framework and serves as a benchmark for evaluating the detection performance of other disease categories.

The mAP_50_ for the “Crack” category performed best among all natural defects, achieving 0.670 and 0.599 on the validation and test sets respectively. As the most common and most damaging type of defect in bridge structures, cracks are visually highly distinguishable due to their linear structure and high-contrast features. Coupled with an ample training dataset, the model is able to fully learn their multi-scale feature representations. Spalling also performed well, with a test set mAP_50_ of 0.373, which even outperformed the validation set's 0.305, indicating that the model exhibits good robustness in extracting the irregular edges and color differences characteristic of spalling defects. For Exposed Rebar, the test set mAP_50_ was 0.304, with precision and recall of 0.420 and 0.334 respectively; these metrics are relatively balanced, owing to the high visual salience of the reddish-brown appearance exhibited by exposed rebar.

However, there is still significant room for improvement in the detection performance of certain categories. The mAP_50_ for the Corrosion category was only 0.059 and 0.049 on the validation and test sets respectively, with recall rates as low as 0.055 and 0.080, making it the worst-performing defect type among all categories. This is because early-stage corrosion typically manifests only as slight color variations and rust stains on the concrete surface; its visual features are ambiguous and highly similar to surface discolouration defects such as Staining, Concrete Leaching, and Efflorescence, making it difficult for the model to establish effective discriminative features. The mAP_50_ for Deep Crack is only 0.062 (validation set) and 0.090 (test set), with recall rates ranging from approximately 0.036 to 0.067, indicating a serious issue with missed detections. Deep cracks share certain visual similarities with ordinary cracks; their distinction relies primarily on subtle differences in crack width and depth. Coupled with the relative scarcity of training samples for this category, this makes it difficult for the model to fully learn the discriminative features. The mAP_50_ for the “Formwork Niche” class is similarly low, at 0.057 and 0.073 for the validation and test sets respectively. This is primarily attributed to the extreme rarity of this class in bridge inspection images, resulting in a severe shortage of training samples.

As shown in Figures 7, 8, the precision–recall and recall–confidence curves reveal that the proposed model achieves excellent and stable detection performance on categories with distinctive features and sufficient training samples, while maintaining high reliability for high-confidence predictions and exhibiting stable training convergence. Nevertheless, a pronounced class imbalance is observed: the curves of most challenging categories remain at a low level and degrade rapidly as recall increases or as the confidence threshold rises, indicating that the model still lacks discriminative power and reliable confidence estimation for low-saliency, small-scale, and long-tail categories. Importantly, the performance bottleneck stems mainly from the difficulty of assigning sufficiently high confidence to correct detections rather than from a deficiency in proposal generation. Consequently, future work should prioritize strengthening the feature representation of hard samples, mitigating class imbalance, and improving confidence

**Figure 7:**
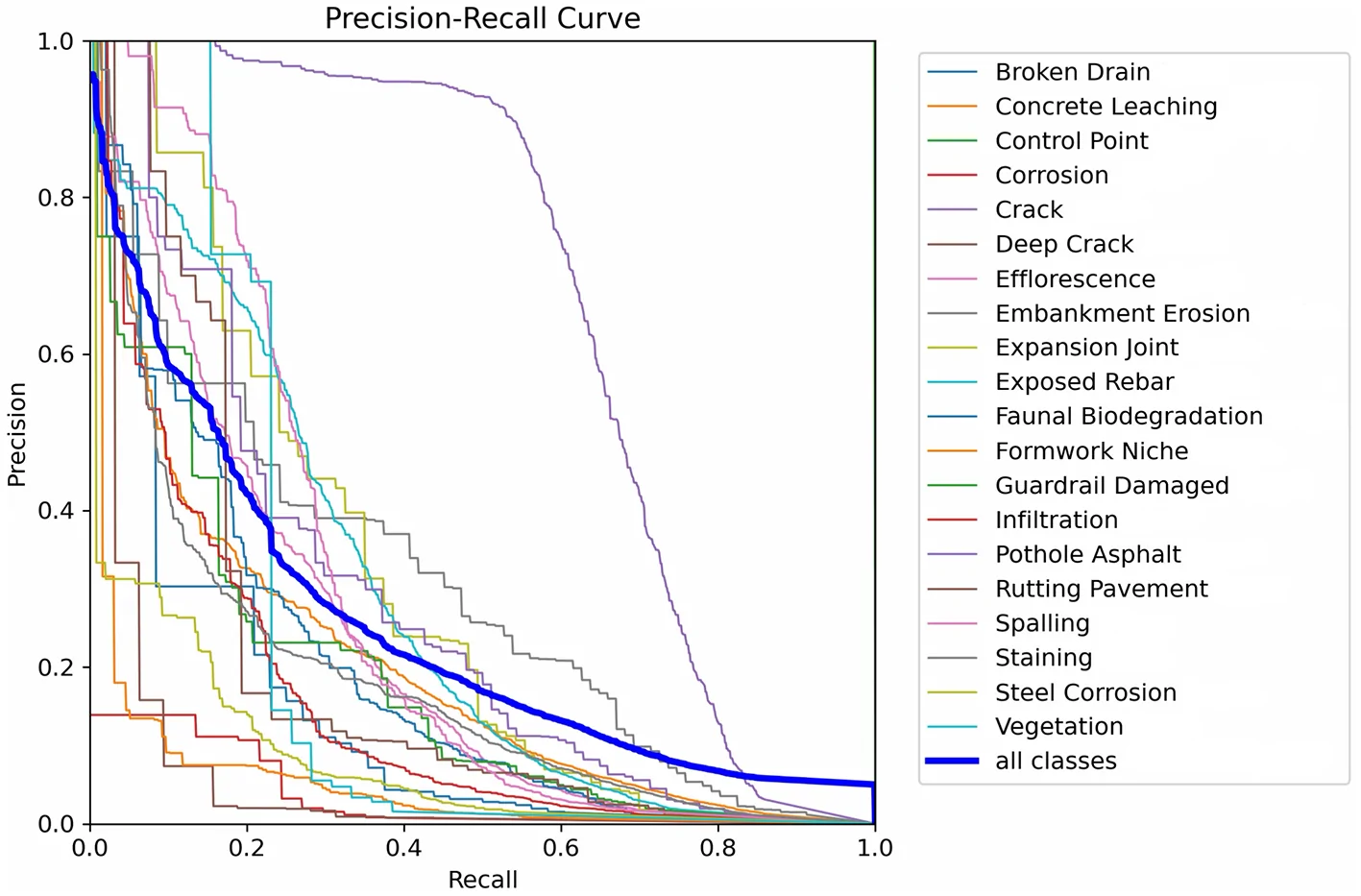
The precision-recall curve.

**Figure 8:**
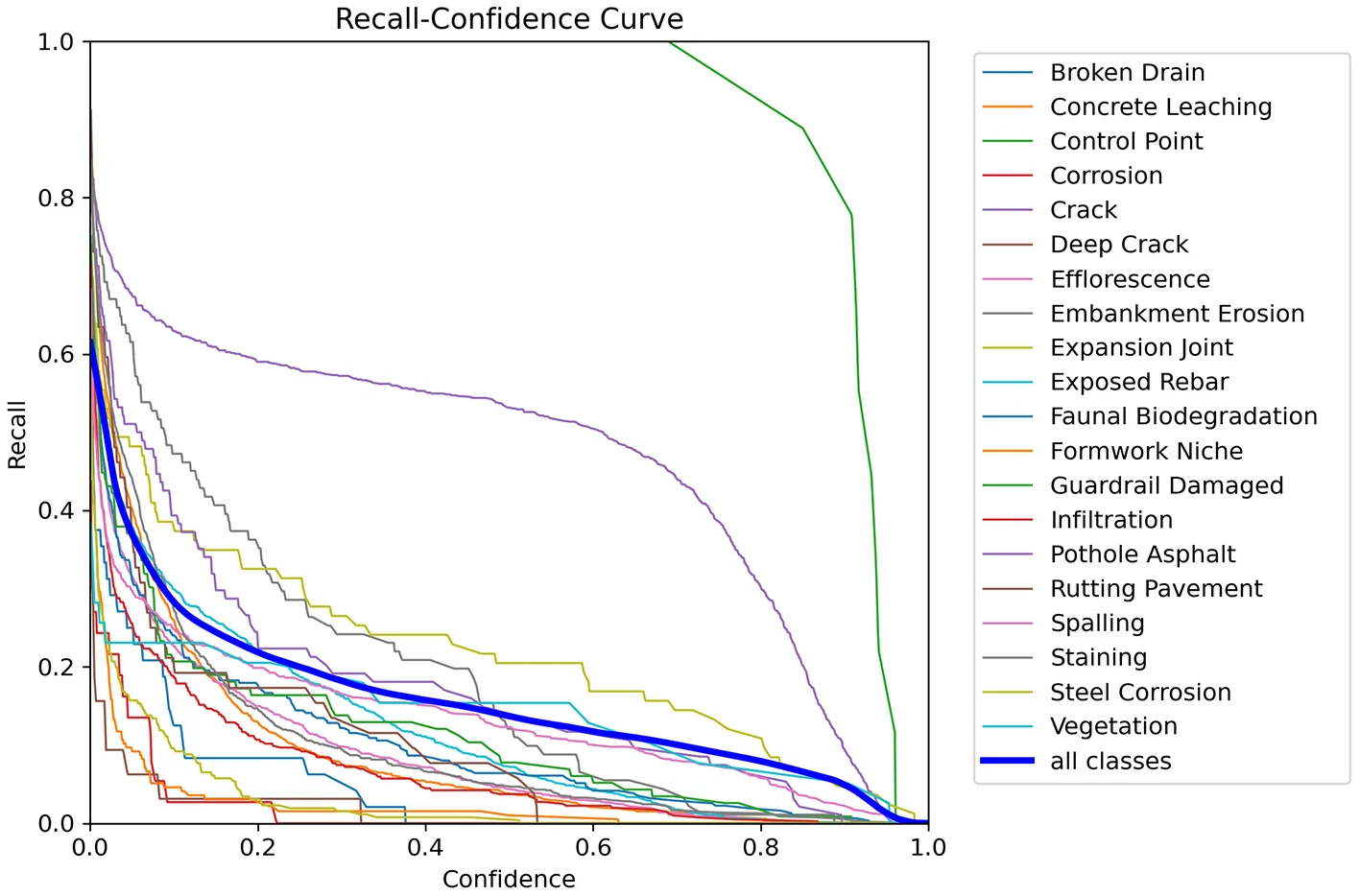
The recall-confidence curve.

calibration, so as to further enhance the overall detection performance and practical applicability of the proposed model.

In terms of the trade-off between precision and recall, different defect categories exhibit distinct characteristics. The “Embankment Erosion” and “Pothole Asphalt” categories display a clear “high recall, low precision,” with recall rates of 0.495 and 0.511 respectively, whilst precision rates were only 0.325 and 0.354. This indicates that the model tends to generate more detection responses for these two defect categories, but also produces a higher number of false positives. This phenomenon can be attributed to the variable morphology of erosion and potholes and the lack of a unified visual template; during the learning process, the model tends to lower the classification threshold to avoid false negatives, at the cost of misidentifying some background textures as defect areas. In contrast, the Spalling, Infiltration, and Exposed Rebar categories exhibit a “high precision, low recall” pattern, with precision reaching 0.483, 0.374, and 0.423 respectively, whilst recall is only 0.286, 0.191, and 0.310 respectively. This indicates that the model possesses a high level of confidence in distinguishing detected defects, yet a significant proportion of actual defects remain overlooked. Such a conservative detection strategy may lead to the omission of safety hazards in engineering applications and requires further improvement through data augmentation, hard sample mining, or category-weighted loss functions.

To address the severely degraded detection performance on the aforementioned minority categories (Corrosion, Deep Crack, and Formwork Niche), we further explore optimization strategies for long-tail distributions. At the data level, Class-Aware Sampler (CAS) or Repeat Factor Sampling (RFS) can be employed to oversample rare categories, while data augmentation techniques such as Copy-Move augmentation and Instance-Balanced Mosaic (IBM) can balance instance sampling across categories during augmentation. At the loss function level, Focal Loss mitigates class imbalance by down-weighting easy examples and focusing on hard, minority-class samples; Class-Balanced Loss designs balanced weights based on the effective number of samples per category; and Class-Weighted Focal Loss further incorporates class frequency information into the Focal Loss formulation. At the model architecture level, decoupled training separates feature learning from classifier learning, while post-calibration methods such as FRACAL calibrate class prediction probabilities during inference, improving rare-class detection without retraining.

As shown in Table 6, our method achieves 172.4 FPS in FP16 mode and 117.6 FPS even in FP32 mode, fully meeting the real-time detection requirements for UAV-based, robotic, or edge-computing bridge inspection scenarios.

**Table 6:** FPS results of our method.

Precision	Inference time (ms)	FPS
FP32	8.5 ms	117.6 FPS
FP16	5.8 ms	172.4 FPS

In summary, our method demonstrates excellent detection performance for classes with distinct visual features and sufficient sample sizes (e.g., Control Point, Crack, and Spalling), whilst facing significant challenges for classes with ambiguous visual features, sparse samples, or high inter-class similarity (e.g., Corrosion, Deep Crack, and Formwork Niche). Future work will introduce fine-grained feature learning mechanisms to enhance the model's ability to distinguish between highly similar defect categories (such as cracks and expansion joints, or corrosion and stains). We will also explore hierarchical detection strategies, whereby defect regions are first detected, followed by fine-grained classification within those regions, to mitigate the impact of inter-class confusion on detection performance.

### 4.4 Comparison of experimental results and analysis

During the inference stage, we follow the official default configuration of the YOLO series, setting the confidence threshold to 0.25 and the NMS IoU threshold to 0.45. This configuration is consistently applied to both the validation and test sets to ensure fair and comparable evaluation conditions. It should be noted that in the subsequent visualization results, we employ a visualization display threshold of 0.3 to filter detection boxes, producing clear and representative detection examples. This display threshold is used solely for visualization purposes and is distinct from both the inference threshold and the evaluation metrics computation; it does not affect any quantitative results reported in this paper.

To comprehensively evaluate the detection performance and computational efficiency of our method, we selected several mainstream object detection algorithms as benchmarks, including the YOLO series (YOLOv3n, YOLOv4n, YOLOv7n, YOLOv8n, and YOLOv12n), Transformer-based detectors (DETR, Deformable DETR, and Swin Transformer) and the classic two-stage detector (Faster R-CNN). All models were compared fairly under identical bridge defect datasets and training conditions; the experimental results are shown in Table 7.

**Table 7:** Performance comparison of different detection models.

Method	Prec.	Rec.	mAP_50_	mAP_50 − 95_	GFLOPs
YOLOv3n (Redmon and Farhadi, 2018)	0.317	0.235	0.198	0.132	1.7
YOLOv7n (Wang et al., 2023)	0.319	0.241	0.211	0.149	2.0
YOLOv8n (Varghese and Sambath, 2024)	0.331	0.267	0.232	0.165	2.2
YOLOv10n (Wang et al., 2024)	0.338	0.278	0.231	0.163	6.0
DETR (Zhu et al., 2020)	0.339	0.273	0.229	0.163	86.0
Deformable DETR (Dai et al., 2017)	0.341	0.269	0.237	0.172	80.0
Swin Transformer (Liu et al., 2021)	0.345	0.293	0.257	0.181	266.0
Faster R-CNN (Girshick, 2015)	0.322	0.238	0.201	0.142	180.0
**Ours**	**0.346**	**0.301**	**0.259**	**0.184**	**8.4**

Overall, our method achieved the best performance across all evaluation metrics, with mAP_50_ and mAP_50-95_ reaching 0.259 and 0.184 respectively, and precision and recall at 0.346 and 0.301 respectively. Compared to the best-performing baseline model, Swin Transformer, our method achieves improvements of 0.8% and 1.7% in mAP_50_ and mAP_50-95_ respectively, whilst significantly reducing computational complexity, thereby achieving an optimal balance between accuracy and efficiency. Compared to YOLOv8n, the best baseline within the YOLO family, our method achieves improvements of 11.6% and 11.5% in mAP_50_ and mAP_50-95_ respectively, with a recall increase of up to 12.7%, demonstrating the effectiveness of the proposed module for the bridge defect detection task.

In the YOLO series of comparative models, detection performance has shown a steady upward trend with each iteration: The mAP_50-95_ of YOLOv3n to YOLOv8n gradually increased from 0.132 to 0.165, indicating that the continuous optimisation of the YOLO architecture in terms of network design, loss functions and training strategies also has a positive impact on the complex task of bridge defect detection. In particular, YOLOv8n, through the introduction of decoupled detection heads and the C2f module, significantly outperforms its predecessors across all metrics. However, the performance of YOLOv12n did not achieve a further breakthrough over YOLOv8n; its mAP_50_ (0.231) and mAP_50-95_ (0.163) are even slightly lower than those of YOLOv8n (0.232 and 0.165, respectively), whilst computational complexity has increased significantly from 2.2 G to 6.0 G. These results indicate that the attention mechanism improvements introduced in YOLOv12n have failed to fully demonstrate their advantages in the specific task of bridge defect detection, instead introducing additional computational overhead. There are other methods for detecting bridge defects, such as YOLODF (Li et al., 2025), YOLOv10-CASPD (Sun et al., 2025), BD-YOLOv8s (Xu et al., 2024), and SFW-YOLO (Luo et al., 2025); however, as these methods do not have publicly available datasets or code, we are unable to compare them with our approach. Building upon YOLOv10n, our method employs SAConv to enhance multi-scale feature extraction, PSAMLLA to facilitate cross-level information interaction, C2fCIB to refine feature representations, and DyHead to optimize detection strategies. With a computational cost of 8.4 G, it achieves performance surpassing all YOLO variants, thereby validating the rationality and effectiveness of the proposed improvement strategies.

Among Transformer-based detectors, Swin Transformer achieved the highest accuracy among all compared models, with mAP_50_ = 0.257 and mAP_50-95_ = 0.181. Swin Transformer achieves an effective balance between local and global feature modeling through its sliding-window self-attention mechanism, and its powerful feature representation capability provides a foundation for the accurate detection of bridge defects. However, its computational cost reaches as high as 266.0 GFLOPs, which is 31.7 times that of our method, constituting a severe efficiency bottleneck in the practical engineering deployment of bridge inspections. The performance of DETR and Deformable DETR is relatively inferior, with mAP_50_ of 0.229 and 0.237 respectively. Although they adopt an end-to-end detection paradigm that eliminates post-processing steps such as NMS, their global self-attention mechanisms incur significant computational overhead on high-resolution bridge images (reaching 86.0 G and 80.0 G FLOPs respectively), and their detection capabilities for dense small objects do not demonstrate a clear advantage in the current task. Our method outperforms all Transformer-based baselines in terms of accuracy whilst maintaining a computational cost of 8.4 G, fully demonstrating the advantages of single-stage detection architectures in the efficiency-accuracy trade-off.

### 4.5 Analysis of visualization results

Figure 9 demonstrates the performance of our method in identifying different types of defects in the task of detecting visible bridge defects. As shown in the figure, the algorithm can accurately detect various typical defects, such as “cracks,” “formwork grooves,” “vegetation,” and “spalling,” and exhibits good localization accuracy under different lighting, texture, and structural conditions. For example, in crack detection, the model generates continuous and well-fitted bounding boxes even for slender, irregularly oriented cracks, with confidence scores generally above 0.5. In easily confused regions such as “formwork grooves” and “vegetation,” the algorithm correctly classifies these features and provides reasonable confidence scores, with no significant false positives observed. Overall, the results in Figure 9 validate the effectiveness and generalization capability of our proposed method in the recognition of multi-class, multi-form defects, demonstrating its potential for deployment in real-world bridge inspection scenarios.

**Figure 9:**
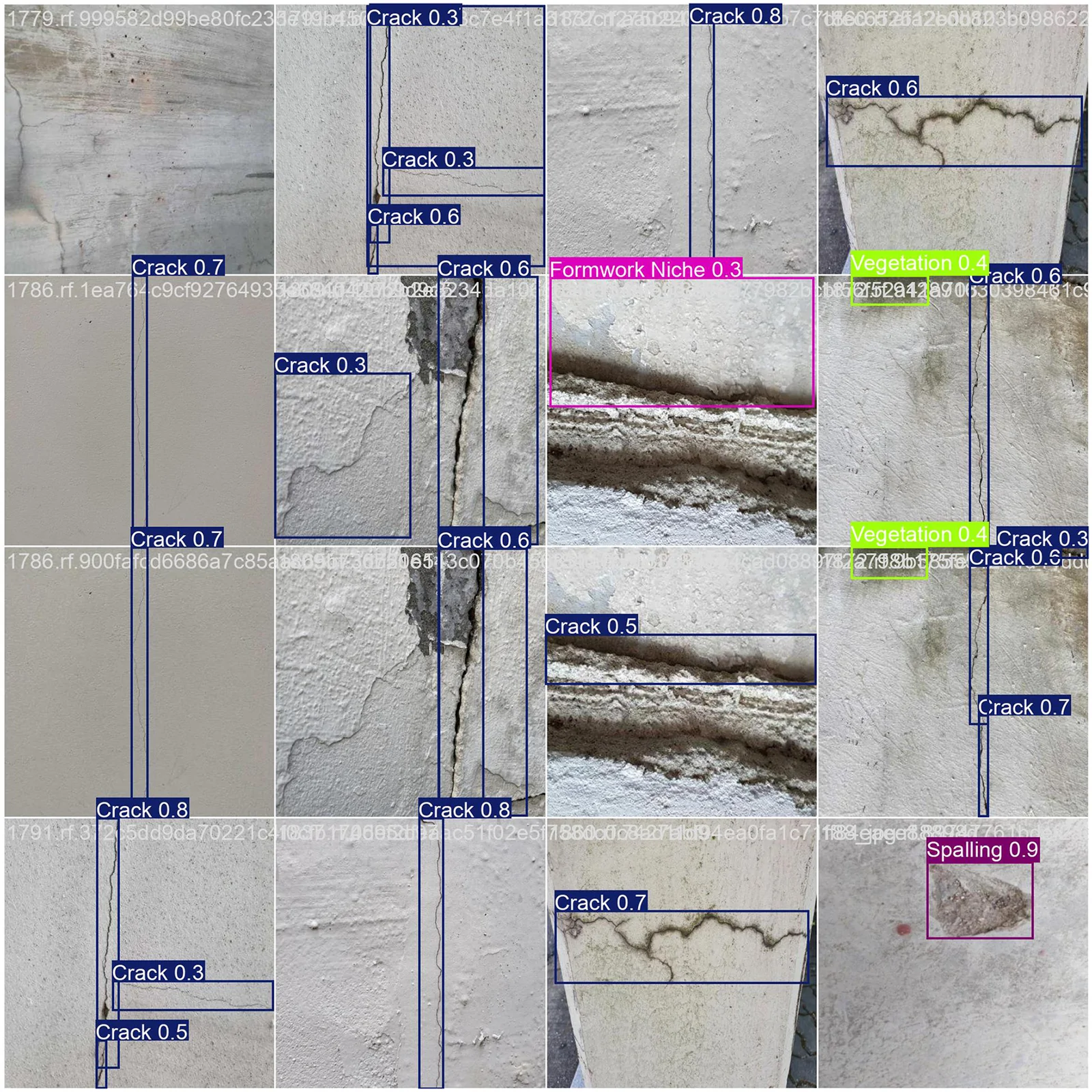
Visualization results of our method on the validation set. For clear visualization, detection boxes with confidence scores below 0.3 are filtered out; this threshold is used solely for display purposes and is distinct from the inference threshold (0.25) used for evaluation.

Figure 10 demonstrates the multi-scale defect detection performance of our method in complex scenarios. As shown in Figure 10, the algorithm can effectively identify defects of varying scales, ranging from fine cracks to large-area spalling. It can also accurately locate and distinguish instances of densely distributed small targets (such as categories 3 and 9 in the third row and second column), demonstrating strong multi-scale feature extraction capabilities. Furthermore, even under complex conditions such as uneven lighting and textural interference, the model maintains high detection stability without significant missed or false detections, validating its robustness in practical applications.

**Figure 10:**
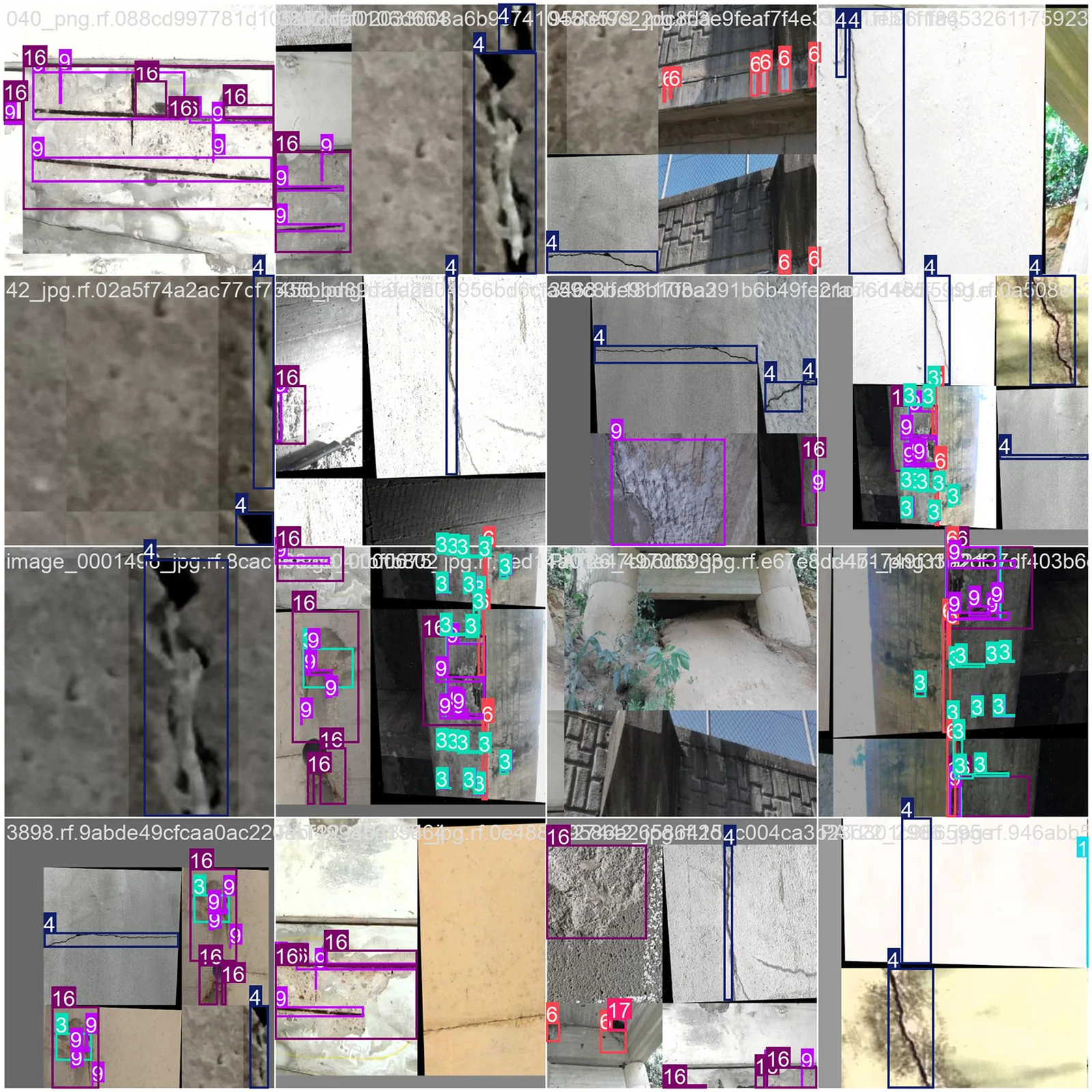
Visualization results of our method on the test set. For clear visualization, detection boxes with confidence scores below 0.3 are filtered out; this threshold is used solely for display purposes and is distinct from the inference threshold (0.25) used for evaluation.

## 5 Conclusion

We propose a method for bridge defect detection based on dynamic receptive field adjustment and multi-scale feature enhancement, which is also highly applicable to underwater and marine infrastructure inspection. In the backbone network, we utilize YOLOv10-n as the base architecture and introduce the C2f_SAConv module, which employs switchable dilated convolutions. By adaptively adjusting the convolution's dilation rate, the network is able to dynamically capture defect features across different receptive field scales without significantly increasing computational overhead. Concurrently, the introduction of the SCDown module in the downsampling stage, in place of standard stride convolution, effectively mitigates the loss of fine-grained defect information during pooling and downsampling. At the end of the backbone network, we innovatively integrate the PSAMLLA module, which utilizes a partial self-attention mechanism to facilitate cross-level information interaction across feature channels and spatial dimensions, significantly enhancing the model's ability to represent low-contrast cracks and blurred lesion boundary features. In the detection head, we introduce the C2fCIB module, which further refines deep semantic features through a contextual information bottleneck structure, aiding in the differentiation of highly similar defect categories. Ultimately, the DyHead detection head achieves efficient localization and classification of bridge defect targets through the dynamic integration of attention mechanisms and scale-aware features.

In summary, our method achieves high-precision identification of various defect types—including cracks, spalling, exposed reinforcement, water seepage, and corrosion—in bridge defect detection tasks. It effectively overcomes core challenges in real-world inspection scenarios, such as complex backgrounds, drastic changes in illumination, and a wide range of defect scales. Beyond the current bridge-surface scenario, the proposed techniques may offer conceptual insights for future exploration in underwater and marine environment perception, where robust visual detection under degraded conditions is equally critical. However, we emphasize that such underwater applications would require dedicated experimental validation on appropriate underwater datasets and imaging models, which we leave as an important direction for future work. Future research will also explore model lightweighting, edge deployment, and integration with multimodal sensing systems to facilitate real-world engineering adoption.

## Data Availability

Publicly available datasets were analyzed in this study. This data can be found here: https://universe.roboflow.com/.
